# Automatic recognition of cephalometric landmarks via multi-scale sampling strategy

**DOI:** 10.1016/j.heliyon.2023.e17459

**Published:** 2023-06-20

**Authors:** Congyi Zhao, Zengbei Yuan, Shichang Luo, Wenjie Wang, Zhe Ren, Xufeng Yao, Tao Wu

**Affiliations:** a College of Medical Imaging, Jiading District Central Hospital Affiliated Shanghai University of Medicine and Health Sciences, Shanghai, 201318, China; b College of Health Science and Engineering, University of Shanghai for Science and Technology, Shanghai, 200093, China

**Keywords:** Deep learning, Orthodontics, Cephalometric landmarks, Automatic recognition

## Abstract

The identification of head landmarks in cephalometric analysis significantly contributes in the anatomical localization of maxillofacial tissues for orthodontic and orthognathic surgery. However, the existing methods face the limitations of low accuracy and cumbersome identification process. In this pursuit, the present study proposed an automatic target recognition algorithm called Multi-Scale YOLOV3 (MS-YOLOV3) for the detection of cephalometric landmarks. It was characterized by multi-scale sampling strategies for shallow and deep features at varied resolutions, and especially contained the module of spatial pyramid pooling (SPP) for highest resolution. The proposed method was quantitatively and qualitatively compared with the classical YOLOV3 algorithm on the two data sets of public lateral cephalograms, undisclosed anterior-posterior (AP) cephalograms, respectively, for evaluating the performance. The proposed MS-YOLOV3 algorithm showed better robustness with successful detection rates (SDR) of 80.84% within 2 mm, 93.75% within 3 mm, and 98.14% within 4 mm for lateral cephalograms, and 85.75% within 2 mm, 92.87% within 3 mm, and 96.66% within 4 mm for AP cephalograms, respectively. It was concluded that the proposed model could be robustly used to label the cephalometric landmarks on both lateral and AP cephalograms for the clinical application in orthodontic and orthognathic surgery.

## 1 Introduction

The identification of anatomical landmarks in cephalometrics is crucial in the treatment planning of orthodontic and orthognathic surgery [1]. Commonly, the orthodontists use information of cephalometric landmarks to assess the relationships between the cranial base and the maxilla or mandible, between the alveolar bone and the maxilla or mandible, and to develop treatment plans [2].

Since 1931, cephalometric analysis did become one important technique for orthodontic diagnoses [3,4]. Cephalometric analysis usually measures the relevant point and line landmarks on the X-ray cephalograms to analyze the morphology, size, location and correlation between the soft and hard tissues of the craniofacial dentition [5,6]. The identification of landmarks significantly affects the accuracy of the cephalometric measurement, and it has been widely used to evaluate the oral condition and to make treatment plans in orthodontics.

Earlier, the cephalometric landmarks were manually labeled and calibrated by experienced trained clinicians, which may lead to measurement errors due to cumbersome procedures [7]. The automatic cephalometric analysis could reduce the labor burden and time expense of orthodontists during treatment planning [8,9]. As a result, a variety of automatic recognition algorithms of cephalometric landmarks had been developed by many researchers [10]. It was categorized as two types: conventional image processing (CIP) and deep learning (DL) approaches [11].

The CIP approach usually extracts the contours of location of the landmark based on priori-knowledge. A proposed automatic recognition algorithm [12] used the prior knowledge to detect the contour shape of craniofacial structures in the predetermined order, after extracting its relevant edges. Additionally, one resolution pyramid was implemented to this algorithm, and the recognition time of the landmarks was obviously shortened with the accuracy of 44% within 2 mm on 5 X-ray images [13]. The median filtering was used to optimize the mentioned resolution pyramid and achieved the success detection rates (SDR) of 95% within 4 mm for 17 landmarks cephalometric analysis [14]. Further, the template matching appended with operators of image edge detection and contour segmentation improved the accuracy of landmark identification to 90% for 17 landmarks on 20 images [15]. Also, the affine alignment was applied to estimate the location of the landmarks by searching the histogram-based boundary to achieve the SDR of 71% and 88% within the 2 mm and 4 mm, respectively [16].

Furthermore, the conventional machine learning (ML) algorithms of CIP were applied to recognize the cephalometric landmarks. The random forest that employed the Haar-like features achieved an SDR of 87.68% within 4 mm on 100 images [17]. Moreover, the random forest with global optimal graph structure obtained an SDR of 80.68% within 4 mm on 100 test images [18]. The independent binary pixel classification achieved an SDR of 70.26% within 2 mm and 88.53% within 4 mm on 100 test images [19]. Also, the random forest regression for the detection of 2D cephalometric markers received the SDR of 77.79% within 4 mm on 100 test images [20].

Recently, the DLs have been proposed in the cephalometric landmarks recognition. The novel convolution neural network (CNN) was used to identify 13 landmarks with an obtained averaged error of 1.24 mm in 950 images [21]. Besides, an improved U-Net network identified 7 landmarks with the SDR of 65.1% within 2 mm, and 84.6% within 4 mm [22]. Another DL network based on landmark recognition obtained a point-to-point error of 1.37 ± 1.79 mm, and the successful identification rate of 88.43% [23]. The R–CNN based CephaNet was proposed that obtained the accuracy of 72.4% and 85.9% within 2 mm and 4 mm, respectively [24]. Compared with other DLs, the YOLOs demonstrated powerful capabilities in the automatic recognition of cephalometric landmarks for clinical applications [25,26]. This was because that the YOLOs preserved fast computation and generalization performance across different object classes and settings, and it has arisen the interests of many researchers for the recognition of cephalometric landmarks [27,28].

Wholly, the CIP method relies on the priori knowledge and it identifies only limited landmarks with low accuracy. Also, it is very easily influenced by the image quality and clinical environments. On the other hand, the recognition using conventional ML methods always require robust models for uncertain sample size in actual applications. In contrast to the CIP approaches, the DLs have the advantages for the betterment of automation and accuracy in the detection of cephalometric landmarks. However, the existing DL methods need to be improved in terms of accuracy and robustness according to data quality and still need more validation on different types of cephalometric landmarks.

With the aim to improve the accuracy and robustness, an automatic recognition approach, namely multiple-scale YOLOV3 (MS-YOLOV3), was proposed to identify the lateral and anterior-posterior (AP) cephalometric landmarks in our study. It was characterized by implementing 4-scale detection modules and spatial pyramid pooling (SPP) module to enhance the feature extraction via fusing shallow and deep features, thereby improving landmarks recognition accuracy. The experimental results proved that MS-YOLOV3 had robust ability in lateral and AP landmarks identification.

## 2 Materials and methods

### 2.1 Experimental data

The present study employed two data sets of lateral and AP cephalograms. One was the public data set which consisted of 400 2D X-ray digital lateral cephalograms [29]. Each image was acquired by the SoredexCRANEX® Excel machine and was also processed by the Soredex SorCom Ceph software. With the archived format in TIFF, the image resolution was set to 1935 × 2400. The second dataset was an undisclosed data set including 163 2D X-ray digital AP cephalograms, which were kindly provided by a domestic incorporated company. Accordingly, each image was acquired by E-ceph software and was archived in the JPEG format with an image resolution of 1900 × 2400. According to the anatomy of maxillofacial structure, the landmarks of lateral and AP cephalograms were manually labeled to 5 × 5 pixels (1 pixels = 0.149 mm) by experienced experts [30]. The present study was approved by our Ethics Review Committee (2021XY06142132197606243519), and informed consent was obtained from all individuals.

### 2.2 Selection of landmarks

**Fig. 1:**
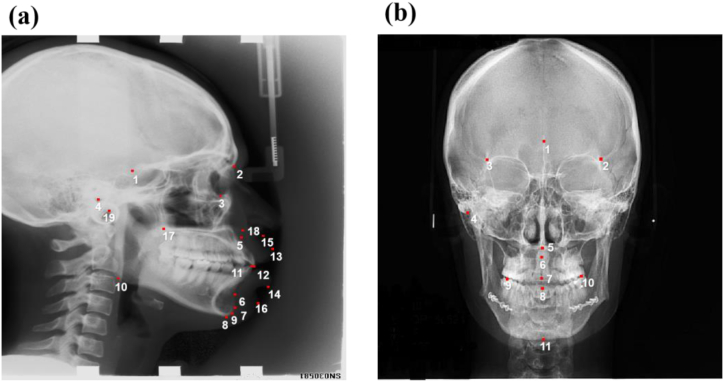
(a) 19 Lateral landmarks; (b) 11 AP landmarks.

For lateral cephalograms, a group of 19 landmarks were selected (Fig. 1(a)), in which 15 hard tissue and 4 soft tissue landmarks were labeled from 1 to 19 for the landmarks of Sella, Nasion, Orbitale, Porion, Subspinale, Supermental, Pogonion, Menton, Gnathion, Gonion, Lower incisal incision, Upper incisal incision, Upper lip, Lower lip, Subnasale, Soft tissue pogonion, Posterior nasal spine, Anterior nasal spine, and Articulate, respectively.

In AP cephalograms, there were 11 chosen landmarks in hard tissues indicated by the numbers of 1–11 for Sella, Left frontomalare orbitale, Right frontomalare orbitale, Right zygomatic, Anterior nasal spine, Posterior nasal spine, Upper incisal incision, Lower incisal incision, Right first molar, Left first molar, and Menton, as shown in Fig. 1(b).

### 2.3 Data preprocessing

The data prepossessing generally consisted of four steps: image conversion, labeling, data amplification and anchor setting. Initially, the image format was converted from the format of TIFF to JPG with the resolution from 1935 × 2400 pixels to 608 × 608 pixels. Subsequently, the label file of landmarks was converted from the format of TXT to XML and the size of each landmark was labeled to 5 × 5 pixels. Then, each data set was fivefold augmented by the transformation of three rotations of 90°, 180° and 270°, flipping, scaling, respectively. Meanwhile, the labels of each image were also changed during the augmented transformations. The original cephalograms were augmented from 400 images to 2100 images for lateral cephalograms, and 163 images to 1304 images for AP cephalograms. Finally, the K-Means clustering was employed to determine the suitable anchor size for matching with the annotated landmarks (5 × 5 pixels), and decided the appropriate number of cluster centers [31]. The relationship between the number of cluster centers and average intersection over union were calculated for achieving an optimal IOU during the target cluster analysis.

### 2.4 Overview of MS-YOLOV3 network

**Fig. 2:**
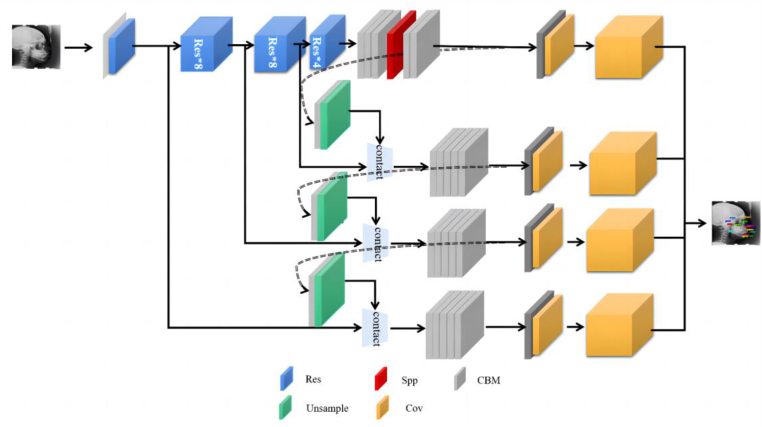
The MS-YOLOV3 architecture. (1) CBM: Convolutional layers, Batch Normalization and Mish activation function; (2) Resn: n stands for number and indicates the number of residual unit; (3) Res unit: residual structure; (4) Concat: tensor splicing; (5) SPP: spatial pyramid pooling.

Based on the YOLOV3 network, the proposed MS-YOLOV3 composed of the basic units of CBM (Convolutional layers, Batch Normalization and Mish activation function) [32], which comprised the convolutional layer, Batch Normalization layer [33], and Mish activation function, as shown in Fig. 2.. Four scale detection modules were implemented by adding the shallow feature detection module in multi-scale feature extraction. Moreover, the SPP module [34] was applied to enhance the deep feature extraction detection module for the fusion of shallow and deep features.

Our improved network contained 118 layers, including the first 74 layers for feature extraction (53 convolutional layers and 21 residual structure layers), and the second 44 layers for scale detection. There were four scales of feature extraction in order to make the complete use of the deep and shallow features. At the first scale, the output feature scale was 13 × 13 with the implemented SPP module; the feature scale increased twice from 13 × 13 to 26 × 26 at the second scale, and the 85th feature layer was fused with the 61st feature layer via route layer. For the third scale, the feature scale increased from 26 × 26 to 52 × 52 and the 97th feature layer was fused with the 36th feature layer via route layer. According to the fourth scale, the output feature scale increased from 52 × 52 to 104 × 104, and the 109th layer was fused with the 11th layer via the route layer. Additionally, the ReLu activation function was used to avoid the problem of gradient over-fitting [[35], [36]].

### 2.5 Experimental setup

**Fig. 3:**
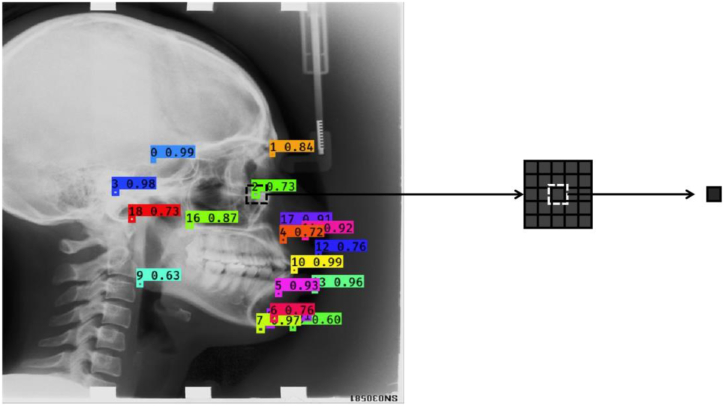
Demonstration of labeled landmarks.

To validate the performance, our method was compared with the classical YOLOV3 on the two lateral and AP data sets. The sizes of training and testing data sets were 1950 and 150 for the lateral data set, and 1154 and 150 for the AP data set, respectively. The main training parameters included: batch size = 10, weight decay factor = 0.001, initial learning rate = 0.001, number of iterations = 2000, learning rate = 0.001, and final loss = 0.0034. The experiment was performed on the platform of LINUX system of CENTOS 7.4.1708 with the GPU configuration of NVIDIA Tesla P100. The center of each labeled boxes of the predicted or true landmarks is demonstrated in Fig. 3. For each label’s indication, the fist number represented the order of label, and the second stood for the confidence level.

The experimental results were qualitatively evaluated by two experienced dentists. Moreover, two metrics of success detection rate (SDR) and mean radial error (MRE) were quantitatively formulated as follows.

(1) SDR: It measures the number ratio of predicted landmarks and actual landmarks within the defined range and was formulated as follows:

(1) $$SDR=\frac{\#\left\{i:\|f_{d}\left(j\right)-f_{r}\left(j\right)\|<H\right\}}{\#\varphi }\times 100\%$$

where, $f_{d}$ and $f_{r}$ represents the coordinates of predicted and actual landmarks, H denotes the error range of 2, 3 and 4 mm, and #φ denotes the number of test images, respectively.

(2) MRE: It defines the averaged Euclidean distance error between predicted and actual landmarks. Here, SD reflects the predicted deviation of all landmarks. It was defined as follows:

(2) $$MRE=\frac{\sum_{i=1}^{N}R_{i}}{N}$$

(3) $$R=\sqrt{{\left(x_{1}-x_{0}\right)}^{2}+{\left(y_{1}-y_{0}\right)}^{2}}$$

(4) $$SD=\sqrt{\frac{1}{N-1}\sum_{i=1}^{N}{\left(R_{i}-MRE\right)}^{2}}$$

where, $R_{i}$ is the Euclidean distance between each pair of predicted and labeled landmarks, N indicates the number of test images, ($x_{1}$, $y_{1}$) denotes the coordinate of each predicted landmark, and ($x_{0}$, $y_{0}$) denotes the coordinate of each labeled landmark.

## 3 Results

### 3.1 Subjective evaluation

**Fig. 4:**
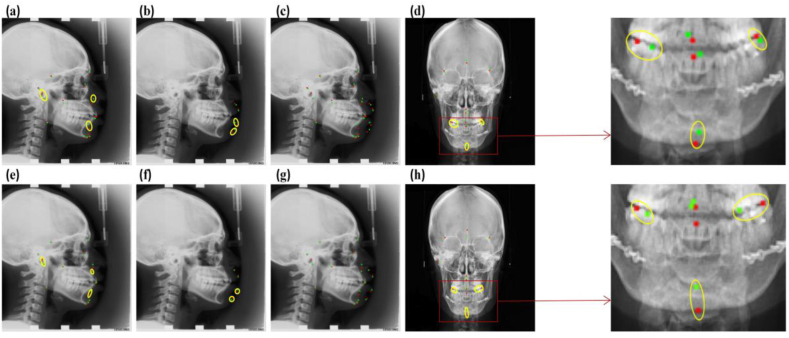
Comparison of proposed MS-YOLOV3 and YOLOV3, top row is MS-YOLOV3 and bottom row is YOLOV3. (a, e): Lateral landmarks in hard tissues; (b, f): Lateral landmarks in soft tissues; (c, g): 19 lateral landmarks without circling; (d, h): 11 AP landmarks in hard tissues.

Fig. 4(a–h) showed the Euclidean distances (Rs) between the predicted and true landmarks. Here, the predicted and true landmarks were labeled by red and green colors, respectively. Any pairwise landmarks of Rs exceeding 11 pixels was regarded as critical errors which were highlighted within yellow circles. There were three lateral landmarks (Supermental, Pogonion, Gonion) for hard tissues, two lateral landmarks (Lower lip, Soft tissue pogonion) for soft tissues, three AP landmarks (Right first molar, Left first molar, Menton) for hard tissues, respectively. It was evident that the proposed approach demonstrated better performance than YOLOV3.

### 3.2 Quantitative evaluation of SDR

For lateral landmarks, the MS-YOLOV3 showed better performance in the detection of landmarks of hard tissues than those of in soft tissues, as shown in Table 1. The landmarks of upper alveolar seat points (Subspinale), ear points (Porion), mandibular angle points (Gonion), and articulate points (Articulate) showed a low accuracy for the two approaches. In Table 1, the averaged accuracy of two models demonstrated the same trend at the error ranges of 2 mm, 3 mm and 4 mm, respectively..

**Table 1:** Comparison of SDR (%) for 19 lateral landmarks.

Landmarks		2 mm		3 mm		4 mm	
		MS-YOLOV3	YOLOV3	MS-YOLOV3	YOLOV3	MS-YOLOV3	YOLOV3
All	Average SDR	80.84	77.21	93.75	87.40	98.14	93.84
Soft tissues	Upper lip	76.67	72.67	98.33	87.33	99.33	92.67
	Lower lip	94.67	89.33	96.67	94.67	98.67	98.00
	Subsanale	92.00	87.33	97.33	92.67	99.33	96.00
	Soft tissue pogonion	73.33	69.33	94.00	87.33	98.67	94.67
	Average SDR	84.17	79.67	96.67	90.50	99.00	95.33
Hard tissues	Sella	95.33	91.33	99.33	95.33	100	98.33
	Nasion	80.00	75.67	86.67	79.00	97.33	87.67
	Orbitale	80.67	77.33	95.33	84.67	99.33	94.00
	Porion	56.67	58.00	79.33	67.33	94.67	84.00
	Subspinale	62.67	58.33	91.33	76.33	99.33	87.33
	Supermental	82.67	83.00	95.33	91.33	98.67	96.00
	Pogonion	81.33	79.67	96.67	88.33	100	94.33
	Menton	90.67	87.00	98.67	94.33	99.33	96.67
	Gnathion	92.67	81.33	98.67	92.67	99.33	97.33
	Gonion	55.33	52.00	82.67	74.67	93.33	89.33
	Lower incisal incision	90.00	87.33	96.00	94.67	98.67	97.33
	Upper incisal incision	91.33	90.67	98.67	95.33	99.33	98.67
	Posterior nasal spine	95.33	90.67	98.67	96.67	100	98.67
	Anterior nasal spine	85.33	82.67	92.67	89.33	96.67	93.33
	Articulate	59.33	53.33	84.67	78.67	92.67	88.67
	Average SDR	80.00	76.55	93.00	86.57	97.91	93.44

Accordingly, the comparison of SDR for 11 AP landmarks is shown in Table 2. The results clearly indicated that the MS-YOLOV3 had better performance than the YOLOV3.

**Table 2:** Comparison of SDR (%) for 11 AP landmarks.

Landmarks	2 mm		3 mm		4 mm	
	MS-YOLOV3	YOLOV3	MS-YOLOV3	YOLOV3	MS-YOLOV3	YOLOV3
Sella	94.67	91.33	97.33	93.33	99.33	97.33
Right frontomalare orbitale	87.33	85.00	92.67	89.33	97.33	94.00
Left frontomalare orbitale	90.00	84.67	92.00	89.33	95.33	93.33
Right zygomatic	91.33	86.67	93.33	91.33	97.33	94.67
Anterior nasal spine	87.33	81.33	94.00	87.33	98.67	93.33
Posterior nasal spine	86.67	81.33	95.67	86.67	98.67	92.67
Upper incisal incision	91.33	80.67	94.67	88.67	98.67	91.33
Lower incisal incision	93.33	90.00	98.67	94.67	99.33	97.33
Right first molar	63.33	43.33	85.33	70.67	90.67	85.33
Left first molar	65.33	57.33	81.33	74.00	89.33	86.67
Menton	92.67	89.33	96.67	94.67	98.67	96.67
Average SDR	85.75	79.18	92.87	87.27	96.66	92.97

### 3.3 Quantitative evaluation of MRE

Table 3 presents the MREs of MS-YOLOV3 and YOLOV3 for the lateral landmarks. The MREs of MS-YOLOV3 were smaller than those of YOLOV3, except for the two MREs of Sella and Gonion, respectively. The MREs of MS-YOLOV3 and YOLOV3 of Nasion, Porion, Subspinale, Menton, Gnathion, Gonion, Upper incisal incision, Subsanale, Soft tissue pogonion and Upper lip displayed significant differences (P < .05).

**Table 3:** MRE comparison for lateral landmarks.

Landmarks		MS-YOLOV3	YOLOV3	*t*-test
		MRE±SD	MRE±SD	
Hard tissues	Sella	1.76 ± 1.13	1.75 ± 0.95	NS
	Nasion	1.34 ± 1.72	2.56 ± 2.01	*
	Orbitale	1.45 ± 1.84	1.73 ± 1.34	NS
	Porion	1.57 ± 1.93	3.05 ± 2.74	*
	Subspinale	1.73 ± 1.67	3.17 ± 2.01	*
	Supermental	1.92 ± 1.76	2.14 ± 2.01	NS
	Pogonion	1.67 ± 1.13	1.86 ± 1.45	NS
	Menton	1.23 ± 0.72	1.78 ± 1.35	*
	Gnathion	1.45 ± 1.34	2.01 ± 1.53	*
	Gonion	2.43 ± 1.56	1.59 ± 1.02	*
	Lower incisal incision	1.67 ± 1.31	1.73 ± 1.10	NS
	Upper incisal incision	1.32 ± 0.62	1.61 ± 0.93	*
	Posterior nasal spine	1.57 ± 1.03	1.67 ± 1.36	NS
	Anterior nasal spine	1.39 ± 1.09	1.46 ± 0.97	NS
	Articulate	1.57 ± 1.97	1.64 ± 1.17	NS
Soft tissues	Subsanale	1.67 ± 1.25	2.34 ± 1.02	*
	Soft tissue pogonion	1.13 ± 0.62	3.25 ± 1.79	*
	Upper lip	1.54 ± 1.32	2.13 ± 1.84	*
	Lower lip	1.82 ± 1.21	1.78 ± 1.34	NS

NS, Not significant; *P < .05.

Table 4 shows that the MREs of MS-YOLOV3 were smaller than those of YOLOV3 for 11 AP landmarks, respectively. Alike lateral landmarks, our proposed approach presented a robust capability in the detection of AP landmarks. The MREs of MS-YOLOV3 and YOLOV3 showed significant differences (P < .05), except for the two MREs of Anterior nasal spine and Upper incisal incision, respectively.

**Table 4:** MRE comparison for AP landmarks.

Landmarks	MS-YOLOV3	YOLOV3	*t*-test
	MRE±SD	MRE±SD	
Sella	1.13 ± 0.72	1.67 ± 1.12	*
Right frontomalare orbitale	1.34 ± 1.22	1.95 ± 1.39	*
Left frontomalare orbitale	1.41 ± 1.67	2.16 ± 2.13	*
Right zygomatic	0.67 ± 1.56	1.35 ± 1.01	*
Anterior nasal spine	1.21 ± 0.92	1.43 ± 1.32	NS
Posterior nasal spine	1.53 ± 0.72	2.16 ± 1.64	*
Upper incisal incision	1.35 ± 0.95	1.78 ± 2.54	NS
Lower incisal incision	0.64 ± 0.77	1.67 ± 2.12	*
Right first molar	1.96 ± 1.79	3.50 ± 2.85	*
Left first molar	2.41 ± 1.96	2.97 ± 2.63	*
Menton	0.65 ± 0.73	1.57 ± 1.14	*

NS, Not significant, *P < .05.

## 4 Discussion

In the present study, we proposed a robust method of MS-YOLOV3 to detect the landmarks of cephalometric analysis. Our proposed method showed robust performance in the detection of cephalometric landmarks on lateral and AP cephalograms. Firstly, with the betterment of YOLOV3, the combination of shallow and deep features presented more precise feature sets. Secondly, in our experiment, the model extracted more shallow features for training, which greatly improved the accuracy of the target detection. And, the robustness of our model was verified both on publicly available and undisclosed data sets. This improved the robustness of the model and avoided the loss of typical features for small target objects.

Although our model displayed more robust capability in identifying the soft and hard tissue landmarks, there existed some landmarks of Subspinale, Articulate, Gonion, Porion and Soft tissue pogonion with low recognition rate. This was due to the fact that the anatomical positions of these landmarks seriously affected the accuracy of recognition. The subspinale was located in the anterior maxillary curve and inevitably produced an error of recognition due to the low contrast between the area of the location of this landmark and the surrounding tissue. This landmark has been addressed as one of the most common landmarks for misidentification in previous studies [36,37]. Further, the articulate was located at the junction of the mandibular condyle and the external dorsal contour of the temporal bone, and identification for model was affected by the overlapping head holder on the lateral cephalograms [1]. The gonion was the midpoint of the contour connecting the mandibular branch to the body, and its morphological structure often overlapped with the cervical spine structure, thus interfering with the accurate positioning of the model [38]. Meanwhile, the porion was located between the soft tissue of the ear canal or the bone tissue edge, and it was difficult to accurately label or even identify it due to the auricular structure that covered it [39]. Among the soft tissue landmarks, the SDR of soft tissue pogonion was low. Since the soft tissue pogonion was the most anterior point of the soft tissue chin and there was no clear tissue structure at this site, the labeled errors were obvious [40].

Learned from the literature, the previous DL methods of CNN [22], U-NET [41], TCNN [42] achieved the best SDR of 75.37%, 43.80%, 78.62% within 2 mm and 88.25%, 78.42%, 95.58% within 4 mm. It was evident that our proposed method showed the outperformed SDR of 80.84%, 85.75% within 2 mm, and 98.14%, 96.66% within 4 mm, respectively, in both public and undisclosed data set.

There were some limitations in our study. Firstly, it is necessary to validate our proposed model on more large samples of different types of data sets. This would relieve the potential overfitting caused by data prepossessing and ensure the generalization and robustness of our model. Secondly, the ultimate recognition accuracy of cephalometric landmarks was easily affected by annotation errors of training landmarks. In the future, more serious data labeling should be repeatedly checked by experienced experts. Thirdly, more innovated DL models should be tried on the identification of cephalometric landmarks.

## 5 Conclusion

The automatic recognition model of MS-YOLOV3 presented higher accuracy for cephalometric landmarks and it can provide a promising assurance for meeting orthodontic clinical needs and assisting clinical surgery.

## Author contribution statement

Congyi Zhao: Performed the experiments; Wrote the paper.

Zengbei Yuan: Performed the experiments; Analyzed and interpreted the data.

Shichang Luo, Wenjie Wang, Zhe Ren: Analyzed and interpreted the data; Wrote the paper.

Xufeng Yao, Tao Wu: Conceived and designed the experiments.

## Funding statement

This work was supported by the 10.13039/501100001809National Natural Science Foundation of China (Nos 61971275, 81830052, and 82072228) and the grants of the 10.13039/501100012166National Key Research and Development Program of China (2020YFC2008700), and the Shanghai Municipal Commission of Science and Technology for Capacity Building for Local Universities (No. 23010502700).

## Data availability statement

The data that has been used is confidential.

## Additional information

No additional information is available for this paper.

## Declaration of competing interest

The authors declare that they have no known competing financial interests or personal relationships that could have appeared to influence the work reported in this paper.
